# Gender Codes and Aging: Comparison of Features in Two Women's Magazines

**DOI:** 10.1093/geroni/igaa057.1039

**Published:** 2020-12-16

**Authors:** Ann O'Hanlon, Therese Mendez, Melissa Morrissette

**Affiliations:** 1 University of New Orleans, New Orleans, Louisiana, United States; 2 University of New Orleans, New Orleans, LA, Louisiana, United States

## Abstract

Magazines and other media promote beauty standards and gender roles in feature articles and advertising. Publications present idealized images of women often in contrast to the average reader’s appearance. Analyses of such images suggest gender roles are reinforced through subtle cues embedded in hand gestures, eye gaze, head posture, and body position (Goffman, 1976). This study analyzed a recurrent feature in two different magazine presenting an idealized standard of aging to mature women. The first magazine, MORE, featured mature women, typically between the ages of 40 and 60, with the banner of “This is what (woman’s age) looks like.” MORE magazine is no longer in press, but another magazine, Women’s Day, began a similar recurrent column featuring a women between 40 and 60 with the title “Own Your Age—Yes, I am (women’s age).” Both features included copy describing the woman’s perspective on life and aging and a listing of specific beauty products that she uses. These features were analyzed as advertisements, because they promote a message about being a woman of a certain age and the specific products used to achieve that look. Three researchers coded 43 images from MORE magazine and 30 images from Woman’s Day for physical characteristics of aging and evidence of Goffman’s gender codes. Most photos presented women who appeared younger than their stated age. Images showed the presence of Goffman’s gender codes including feminine touch, ritualization of subordination, licensed withdrawal, and infantilization and were more prevalent in the MORE feature than Woman’s Day column.

